# PDAC as an Immune Evasive Disease: Can 3D Model Systems Aid to Tackle This Clinical Problem?

**DOI:** 10.3389/fcell.2021.787249

**Published:** 2021-12-10

**Authors:** Shruthi Narayanan, Silve Vicent, Mariano Ponz-Sarvisé

**Affiliations:** ^1^ Clinica Universidad de Navarra, Medical Oncology Department, Pamplona, Spain; ^2^ Program in Solid Tumors, Center for Applied Medical Research, University of Navarra, Pamplona, Spain; ^3^ IdiSNA, Navarra Institute for Health Research, Pamplona, Spain; ^4^ Centro de Investigación Biomédica en Red de Cáncer (CIBERONC), Madrid, Spain; ^5^ Department of Pathology, Anatomy and Physiology, School of Medicine, University of Navarra, Pamplona, Spain

**Keywords:** PDAC, immunotherapy, immune evasion, 3D model systems, co-culture

## Abstract

Pancreatic ductal adenocarcinoma (PDAC) is an aggressive cancer with a high mortality rate. The presence of a dense desmoplastic stroma rich in fibroblasts, extracellular matrix, and immune cells plays a critical role in disease progression, therapy response and is a distinguishing feature of PDAC. PDAC is currently treated with a combination of surgery, chemotherapy and radiation therapy in selected cases which results in long-term survival only in a small percentage of patients. Cancer therapies that incorporate immunotherapy-based techniques have become increasingly common in recent years. While such a strategy has been shown to be effective for immunogenic, “hot” tumors like melanoma and lung cancer, thus far PDAC patients display poor responses to this therapeutic approach. Various factors, such as low tumor mutational burden, increased infiltration of immunosuppressive cells, like MDSCs and Treg cells promote tolerance and immune deviation, further aggravating adaptive immunity in PDAC. In this review we will elaborate on the ability of PDAC tumors to evade immune detection. We will also discuss various 3D model system that can be used as a platform in preclinical research to investigate rational combinations of immunotherapy with chemotherapy or targeted therapy, to prime the immune microenvironment to enhance antitumor activity.

## 1 Introduction

Pancreatic ductal adenocarcinoma (PDAC) is an aggressive malignancy with a high mortality rate. Indeed, prognosis for PDAC patients is one of the poorest among all cancers (Dell’Aquila et al., 2020). PDAC is characterized by a rapid progression, a high propensity for metastatic spread and an exceptional resistance to all forms of anticancer treatment (Mizrahi et al., 2020; Park et al., 2021). The 5-year survival rate of PDAC patients has climbed from 6 to 10% between 2014 and 2021 as a result of current therapeutic strategies based on a combination of surgery, chemotherapy and radiation therapy (American Cancer Society, 2021). However, the long-term survival benefit occurs only in a small percentage of patients. Thus, even though this moderate improvement in survival rates demonstrates progress, there is still a pressing clinical need to improve patients’ outcome for this devastating disease.

At the histopathological level, PDAC presents with a prominent desmoplastic stroma, which consists of a heterogeneous cell microenvironment that includes fibroblasts, immune and endothelial cells, as well as a rich extracellular matrix of collagen and non-collagen proteins, such as laminins, fibronectin and other glycoproteins (Santi et al., 2018; Rawla et al., 2019). Such dense stroma represents not only a physical but a biologically functional barrier that limits infiltration and antitumor activity of immune cells as well as proper diffusion of therapeutics, therefore playing a critical role in disease progression and therapy response.

At the genomic level, multiple genetic and epigenetic alterations characterize PDAC. A prevailing genomic feature of PDAC is the high rate of KRAS mutations, found in ∼90% of cases (Hezel et al., 2006). Mutations in KRAS occur early in PDAC tumorigenesis and function as an initiating event of the disease (Biankin et al., 2012; Waddell et al., 2015; Witkiewicz et al., 2015; Frappart and Hofmann, 2020). Despite the sequential acquisition of additional genomic alterations that contribute to mold the course of PDAC development (Schneider et al., 2017), KRAS mutations strongly influence tumor maintenance and metastasis (Collins et al., 2012). Thus, KRAS oncoprotein stands as a key molecular target in this malignancy, particularly in the context of advanced disease where therapeutic options are required. Unfortunately, neither targeted therapies against canonical KRAS effectors nor the most recently developed KRAS inhibitors targeting the G12C mutation, which only occurs in 2–3% of all cases, have demonstrated significant benefit for PDAC patients, what emphasizes the need for novel treatment options (Bryant et al., 2014; Brown et al., 2020).

Large-scale analysis of the PDAC genome has revealed remarkable inter- and intra-tumoral heterogeneity and complexity. Several studies on transcriptional profiling of patient PDAC specimens have indicated the existence of multiple tumor subtypes, each with distinct molecular characteristics. Existence of classical and basal-like subtypes have been validated across multiple studies in both primary and metastatic samples (Bailey et al., 2016; Raphael et al., 2017; Cao et al., 2021; Flowers et al., 2021). Classical tumor subtype is characterized by the expression of epithelial markers, whereas basal-like subtype present with more mesenchymal features like the expression of laminin and basal keratin, stem cell and epithelial-to-mesenchymal transition (EMT) markers. The importance of such a classification lies in the fact that the basal subtype tumors are poorly differentiated and correlate with worse prognosis and drug response (O’Kane et al., 2020). However, the complex mechanisms underlying the establishment of a specific subtype are still under investigation. The stromal compartment is another source of intratumoral heterogeneity. Within the tumor microenvironment (TME), several subpopulations of fibroblasts and macrophages can be identified (Elyada et al., 2019). Cancer associated fibroblasts (CAF) are a diversified population of cells with the capacity to modify the TME and influence the fate of tumor cells (Sahai et al., 2020). In PDAC, transcriptionally distinct macrophage subpopulations arise from various sources, including embryonic precursors, adult hematopoietic stem cell (HSC) progenitors, and monocytes (Poh and Ernst, 2021). The presence of macrophages has been negatively correlated with PDAC patient survival (Yu et al., 2019). Furthermore, differential presence and ratio of immune cell populations in the tumor may account for intratumoral heterogeneity. Taken together, there is considerable evidence that these diverse stromal populations play a pivotal role in tumor development, ECM remodeling, and therapy response.

This review provides an overview of factors responsible for immune evasion in PDAC that leads to failure of immunotherapy. We also discuss emerging 3D preclinical models that can be utilized in developing effective treatment strategies.

## 2 Failure of Immunotherapy in Pancreatic Ductal Adenocarcinoma

Cancer treatments that incorporate immunotherapy-based techniques have revolutionized the Oncology field in recent years. However, patient responses vary dramatically across cancers (Nixon et al., 2018). For instance, while immunotherapy has become standard of care in melanoma or lung adenocarcinoma, it has so far been ineffective in some gastrointestinal tumours including PDAC, which is particularly refractory to immune-based therapeutic strategies. Pembrolizumab, an FDA-approved drug that targets the PD-1/PD-L1 pathway for the treatment of solid tumors with a high mutation burden, as well as tumors with high microsatellite instability (MSI-H) or mismatch repair deficiency (dMMR), can only be used in the 1–2% of PDAC patients who have these characteristics (Luchini et al., 2021). A multiparameter analysis of the immune landscape in PDAC revealed heterogeneous expression of immune checkpoint receptors in individual patients’ T cells and increased markers of CD8^+^ T cell dysfunction in the disease stage (Steele et al., 2020). This suggests that a one-size-fits-all approach to immune checkpoint inhibitor therapy may not apply to PDAC. Instead, the therapeutic strategies should be tailored to specific individuals based on their checkpoint expression profile, genomic characteristics and TME populations’ profile such (i.e., lymphocyte infiltration). Overall, the use of immunotherapy in PDAC could be improved with the design of rational combinations with chemotherapy and, in this regard, research regarding the unique biology of PDAC should be explored further.

## 3 Factors Responsible for Failure of Immunotherapy in Pancreatic Ductal Adenocarcinoma

**FIGURE 1:**
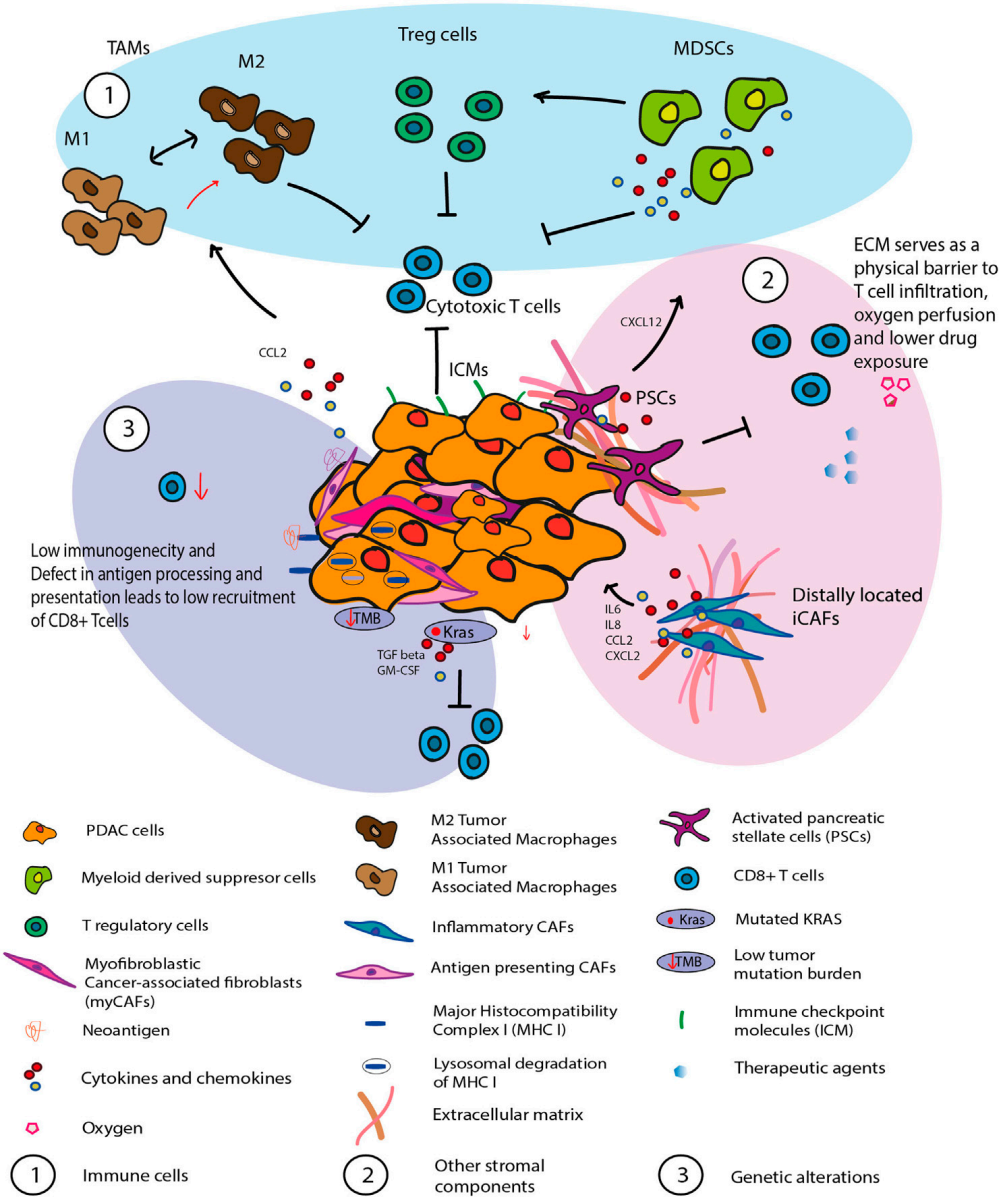
Illustration of the immune evasive and immune suppressive PDAC tumor microenvironment. The interaction between the tumor and the other cellular components of the TME culminate in a very complex immunosuppressive TME. 1) Immune cells such as MDSC, TAM, Treg are implicated in immune evasion and tumor growth in PDAC 2) Other stromal components such as PSCs and inflammatory CAFs has been shown to contribute towards T cells dysfunction. The desmoplastic ECM which is a major component of the PDAC stroma forms a physical barrier which prevents T cell infiltration as well as effective drug exposure. 3) launch of an appropriate immune response is compromised by tumor cell-inherent resistance mechanisms which include tumor mutational load and abnormal expression of oncogenic signatures (i.e., KRAS). Lower level of quality neoantigen and defect in antigen processing and presentation also leads to low recruitment of CD8^+^ T cells to the tumor site.

Inactivation of the immune response by the immune suppressive TME, as well as impaired effector T cell infiltration contribute to the poor prognosis of PDAC patients. The particular host tissue distinguishes the response of PDAC to immunotherapy from that of other solid cancers. PDAC features an abundance of tumor stroma, where distribution and activity of different immune cell populations are governed by its interactions with other cellular components of the TME and the tumor (Feig et al., 2012). These interactions culminate in a very complex immunosuppressive TME. Here, we outline the key factors responsible for the poor therapeutic response, focusing on the immune cell network around cancer cells, additional stromal components, and tumor intrinsic mechanisms (Figure 1).

### 3.1 Immune Cells

PDAC tumor microenvironment shows a highly heterogeneous immune infiltration profile in individual patients (Chakrabarti et al., 2018). In early stages, PDAC’s TME is distinguished by the lack of evidence for T cell activation due to its strongly immunosuppressive traits (Stromnes et al., 2017). As the disease progresses, a subset of patients with unresectable late stages of disease had a profile of CD8^+^ T cells with a more pronounced exhaustion signature (Huber et al., 2020; Steele et al., 2020). By definition, T-cell exhaustion is a T-cell differentiation state caused by persistent antigen exposure, which activates T-cell receptor (TCR) signaling during chronic infections and increases with age (Wherry and Kurachi, 2015). In PDAC, T cells transform into an exhausted differentiation state, which is characterized by upregulation of inhibitory receptors like PD1 or TIGIT, resulting in loss of effector function (Freed-Pastor et al., 2021). Furthermore, the combinations of immunological checkpoint genes expressed in each patient’s CD8^+^ T cells were distinct, suggesting that immune-modulatory therapies should potentially be targeted to specific individuals based on their tumor checkpoint expression profile (Steele et al., 2020).

Multiple types of tumor-promoting immune cells such as myeloid-derived suppressor cells (MDSCs), tumor-associated macrophages (TAMs) or regulatory T cells (Tregs) infiltrate tumors and enable immune evasion and tumor growth (Martinez-Bosch et al., 2018; Chen et al., 2020). These myeloid cells are attracted from the circulation to the tumor site via chemokine pathways that tumor cells co-opt to enhance myeloid cell attraction like CCL2 (Schmid and Varner, 2010; Gu et al., 2021). Of note, a recent study demonstrated that tumoral MDSCs can stimulate Treg cell proliferation and/or development in a cell-cell dependent way in mouse models (Siret et al., 2020). Furthermore, they discovered that Treg cells influence the survival and/or proliferation of MDSCs in PDAC. Paradoxically, another study using murine models found that reducing Tregs did not improve immunosuppression, but rather promoted tumor growth (Zhang et al., 2020). The authors observed Treg cell depletion reprogramed the fibroblast population, with loss of tumor-restraining, smooth muscle actin-expressing fibroblasts (myCAFs), similar to what was described in a previous study (Rhim et al., 2014). Interestingly, Zhang et al. (2020) also observed an increase in chemokines Ccl3, Ccl6, and Ccl8, which resulted in enhanced myeloid cell recruitment, immune suppression and tumor progression. TAMs are one of the most abundant immune population in the TME. TAMs can originate from either monocytes or tissue-resident macrophages of embryonic origin (Zhu et al., 2017). They can further differentiate into functionally distinct M1 and M2 macrophages depending on the polarizing signals present in the microenvironment. M1 macrophages are known to be pro-inflammatory with anti-tumor activity, whereas M2 macrophages secrete anti-inflammatory signals aiding tumor progression (Lankadasari et al., 2019). TAMs have a well-recognized role of immune suppression as evidenced by a study lead by Nywening (Nywening et al., 2018). They reported that targeting CCR2^+^ TAMs along with tumor associated CXCR2^+^neutrophils (TAN) launched a robust antitumor immune response as well as better chemotherapeutic response in PDAC. Another interesting study using orthotopic and genetically engineered mouse models of PDAC found that PI3Kγ selectively drives immunosuppressive transcriptional programming in macrophages inhibiting adaptive immune responses and promotes tumor cell invasion and desmoplasia (Kaneda et al., 2016).

The existence of a delicate balance between the populations of CD4^+^ and CD8^+^ subsets determines whether the environment is anti- or pro-tumorigenic (Clark et al., 2007; Saka et al., 2020). Notably, regulating the differentiation of naïve CD4^+^ T cells into Th1, Th2, Th17, Th9, Th22, and Tregs is essential for eliminating immunosuppressive restrictions from the tumor environment and boosting effector T-cell activity (Knochelmann et al., 2018). It is possible that the disruption of the correct ratio of these cell populations causes immune evasion in cancer and even the failure of several immune cell targeted therapies.

### 3.2 Other Stromal Components

Phenotypically, the dense ECM present in the PDAC composed of collagen I, laminin and hyaluronan (HA) alone accounts for up to 90% of the total tumor volume making up the stromal components (Murphy et al., 2021). Because of their dense tumor architecture, PDAC has poor perfusion compared to normal tissues and even other cancers. Such particular architecture causes a distorted blood vessel network, which obstructs oxygen perfusion and causes hypoxia, which in turn promotes tumor progression (Jacobetz et al., 2013). Such poor tissue perfusion will inevitably result in a significant reduction in total treatment exposure as well affecting its efficacy. Furthermore, immune suppressive myeloid derived cells have been demonstrated to be more infiltrating in such a milieu than lymphocytes, contributing to the failure of numerous immunotherapies. To complicate matters, hypoxia is known to trigger the activation of pancreatic stellate cells (PSC), which are thought to be PDAC’s ‘‘partners in crime’’ (Yamasaki et al., 2020).

PSCs secrete a variety of soluble cytokines that has been shown to contribute towards T cell exhaustion and dysfunction (Ho et al., 2020). Activated PSC are known to regulate T-cell migration. They sequester anti-tumor CD8^+^ T-cells, preventing them from infiltrating juxtatumoral stromal compartments and therefore limiting access to cancer cells (Ene-Obong et al., 2013). Furthermore, they have been shown to recruit myeloid-derived suppressor cells (MDSCs) to the tumor site *via* the CXCL12/CXCR4 axis. Activated PSCs also promote M1 macrophage development into a pro-tumor M2 phenotype (Puré and Lo, 2016). It is well known that PSCs are responsible for producing the desmoplastic ECM within PDAC, such an ECM also forms a physical barrier which prevents T cell infiltration as well as effective drug exposure (Di Maggio et al., 2016; Fu et al., 2018). Cancer-associated fibroblasts (CAFs) originating from activated PSC’s form a major cellular component of the TME. CAFs can be further characterised into functionally distinct subtypes: α-SMA + myofibroblastic CAFs (myCAFs), inflammatory CAFs (iCAFs) and fibroblasts with antigen presenting ability (apCAF) (Pereira et al., 2019). Studies have shown that myCAFs restrain tumor cell growth, whereas iCAFs display a more pro-tumorigenic function. iCAFs secrete inflammatory factors such as interleukin (IL)-6, IL-8, CCL2, and CXCL2 that promote tumor growth and also promote T-cell dysregulation by promoting expression of immune checkpoint inhibitors (PD-1, TIM-3) (Gorchs et al., 2019; Gorchs and Kaipe, 2021).

### 3.3 Genetic Alterations

Antitumor immunity is also hampered by tumor cell-inherent resistance mechanisms, which include tumor mutational load and unusual expression of oncogenic signatures (Tang et al., 2021). PDAC is regarded as a “cold tumor” with a low T cell infiltration and low tumor mutation burden (TMB) with few neoantigens. This makes successful application of immunotherapy in these cancers very difficult. Neoantigens are the consequence of mutations that overwrite the coding sequence and cause proteins to be transcribed that are not present in the normal proteome (Chen et al., 2020). These proteins can activate the immune system and are the basis of cancer immunity. In a recent study in long-term survivors of PDAC, the highest number of quality neoantigen load in combination with abundant CD8^+^ T-cell infiltrates within the tumor correlated with survival (Balachandran et al., 2017). The researchers have also identified MUC16 as apparent neoantigenic hotspot in rare long-term surviving patients. This is an exciting development as there is great potential to harness such neoantigens therapeutically.

Some tumor cells have devised a variety of methods to prevent identification by host immune cells, allowing them to evade immune regulation and continue cancer growth. PDAC cells can evade immune recognition by downregulating expression of antigen processing and presentation molecules, like the major histocompatibility complex (MHC) I proteins, TAP (transporter associated with antigen processing) protein and latent membrane proteins (Pandha et al., 2007; Martinez-Bosch et al., 2018; Hiraoka et al., 2020; Yamamoto et al., 2020). The loss of neoantigens due to the inherent genetic instability of the tumors has also been reported (Mardis, 2019). Another level of immune evasion relates to expression of dominant oncogenic drivers in PDAC. KRAS oncogene imparts its pro-tumoral activity *via* regulation of cell (proliferation, migration, invasion, apoptosis blockade, and metabolic adaptation) and non-cell autonomous (tumor microenvironment remodelling and immune suppression) mechanisms (Zhang et al., 2014). Mutant KRAS induces expression of cytokines such as transforming growth factor *β* (TGFβ) and granulocyte macrophage colony-stimulating factor (GM-CSF) via the classical Raf/MAPK and PI3K signaling pathways (Cullis et al., 2018). These secreted immunomodulatory factors play dominant roles in shaping the immune microenvironment. For instance, KRAS oncogene dependent upregulation of GM-CSF has shown to recruit of Gr1+CD11b + MDSCs and hinder antitumour T cell activity (Pylayeva-Gupta et al., 2012). Another study demonstrates the immune suppressive role of KRAS by its genetic ablation in a mouse model. The authors noted increased influx of immune cells into the tumor and tumor regression upon oncogenic inactivation, and identified BRAF and MYC as key mediators of KRAS-induced immune evasion (Ischenko et al., 2021). The ability of mutant KRAS to modulate tumor immunity highlights the importance of adopting a combinatorial treatment approach with KRAS inhibition and simultaneous stimulation of the immune system.

## 4 3D Models to Investigate Immuno-Oncology in Pancreatic Ductal Adenocarcinoma

**FIGURE 2:**
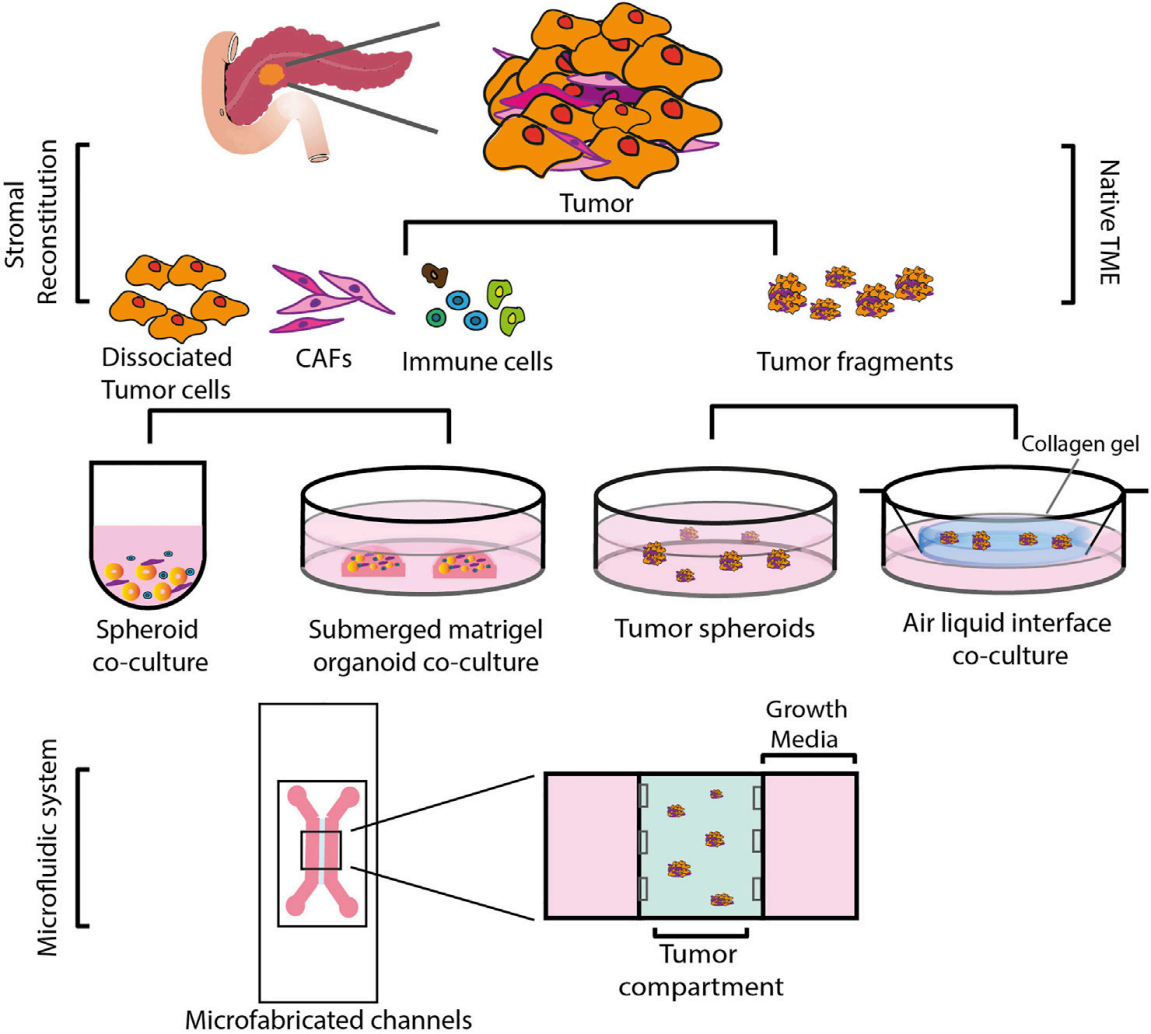
Schematic representation of various 3D co-culture systems. These could be broadly divided into two types: i) Reconstituted TME, in which cells are mechanically and enzymatically dissociated from the primary tumor tissue and sorted and expanded into different cell populations. Tumor cells grown as spheroids or organoids are then reconstituted with stromal cells of choice. ii) Native TME, where primary tumor tissue is mechanically fragmented and grown as tumor spheroids on low attachment plates or cultured in an air-liquid interface, embedded in a collagen gel in an inner transwell dish. The culture media from an outer dish diffuses into the inner dish via a permeable transwell, and the top of the collagen layer is exposed to air via an ALI, allowing cells to oxygenate. Both of these methods can be incorporated into a specifically designed microfluidic system.

Anticancer drug activity has traditionally been assessed in two-dimensionally (2D) cultured cancer cell lines. However, it is now recognized that 2D-cultured cells are incapable of simulating the complex microenvironment of the tumors *in vivo* (Duval et al., 2017). This might be one of the reasons why many drugs proven to be effective in 2D preclinical models failed in the clinic. A large body of research now focuses on the development of alternate, three dimensional (3D) models as a way to overcome some of the drawbacks of 2D-culture models (Figure 2) (Suri et al., 2020). Transplantable mice models in which PDAC cells are injected either orthotopically or ectopically result in tumors that are histologically different from human PDAC, with a higher vascularity and a lower desmoplasia, presenting with increased drug sensitivity (Olive et al., 2009). Genetically modified animal models, on the other hand, more accurately reflect the stroma of PDAC. These models, however, are resource-intensive and time-consuming to develop (Lee et al., 2016). Furthermore, observing tumor progression and its response to treatments over several time periods is challenging in such a model, as studies frequently offer only single endpoint data. Because of these factors, getting mechanistic and temporally resolved data while examining tumor-stromal interactions in PDAC is difficult. In order to better understand cell-stromal interactions and make accurate treatment predictions, 3D models offer a better alternative compared to 2D systems as they more closely recapitulate processes such as cell-cell, cell-matrix interaction, tumor heterogeneity and gradient formation of nutrients, oxygen, and drugs (Table 1). When compared to mouse models, 3D models are considerably more accessible and amenable to genetic manipulation (Heinrich et al., 2021). Here we describe different 3D models developed that have application in immune oncology.

**TABLE 1:** Overview of different 3D organoid coculture system.

Features	Spheroids	Organoids		Microfluidic system
		Reconstituted TME	Native TME	
Cell source	Established cell lines	Patient derived cells, established cell lines	Patient derived tissues	Patient derived cells, established cell lines
Co-culture method	Reconstitution with stromal cells	Reconstitution with stromal cells	Tumor cells, stroma from native tissue - fibroblasts, tumor-infiltrating lymphoid and myeloid cells, including DCs, MDSCs	Reconstitution with stromal cells or maintain stromal components from the native tissue
Advantages	Easy to establish and maintain; captures the essential pathobiology of PDAC, like the presence of hypoxia, nutrient gradient, a necrotic core and soluble factor distribution; can simulate chemoresistance in 3D with a more matrix-rich phenotype	Recapitulates molecular and morphological features of the original tumor; enables study of tumor-stroma interaction; can potentially be used to study patient specific drug response	Recapitulates molecular and histological features of the original tumor; retains stromal components from the native tissue; Long term culture; enables study of tumor–stroma interactions; can be used to study patient specific drug response	Requires small amount of tissue and medium; Both Reconstituted and Native TME organoids can be used; enables study of tumor–stroma interactions; can be used to study patient specific drug response; Can be modified to increase throughput
Disadvantage	Lacks native stromal components; depends on cell self-aggregation, which restricts control over the 3D culture environment and its architecture	Lacks native stromal components; collaboration between the lab and Clinicians needed to obtain patient derived tissues;	Contains only tumor infiltrating T cells and not circulating tumor cells; difficult to visualize tumor-stroma interaction in real time; contacts between the lab and Clinicians needed	Specialized devices are required
References	Longati et al. (2013), Ware et al. (2016), Courau et al. (2019), Nunes et al. (2019); Norberg et al. (2020)	Jenkins et al. (2018), Kopper et al. (2019), Tiriac et al. (2019), Delle Cave et al. (2021)	Ootani et al. (2009), Li et al. (2014, 2016), Neal et al. (2018)	Zervantonakis et al. (2012), Aref et al. (2018), Jenkins et al. (2018), Aung et al. (2020), Palikuqi et al. (2020)

### 4.1 Spheroids

Spheroids are cell aggregates growing in suspension in 3D with or without an extracellular matrix. Unlike 2D models, spheroid models are able to capture the essential pathobiology of PDAC, like the presence of hypoxia, nutrient gradient, a necrotic core and soluble factor distribution (Ware et al., 2016). It is worth noting that the spheroid size can be defined by fine-tuning the technique, making this model extremely reproducible. Spheroids with a diameter of 150 µm have been shown to display cell–cell and cell–matrix interactions, as well as an altered expression profile. A tumor spheroid of size 200–500 µm displays oxygen, nutrition, and other soluble factor gradient development (Hirschhaeuser et al., 2010). At a diameter of >500 μm, cells in the perimeter are actively proliferating, while cells in the interior are quiescent and eventually die by apoptosis or necrosis, resulting in the formation of a necrotic core. Spheroid assays are highly reproducible and relatively low cost (Nunes et al., 2019). Furthermore, they can simulate chemoresistance in 3D with a more matrix-rich phenotype. This experimental approach may be helpful for drug testing as it more closely simulates the *in vivo* scenario (Longati et al., 2013).

Spheroid models that incorporate the tumor cells with stromal components are an attractive model to investigate the efficacy of tumor stroma targeting immunotherapies in the preclinical setting. In a triple co-culture with PDAC and cancer associated fibroblasts, myeloid cell infiltration was observed within spheroid compartment (Kuen et al., 2017). Also, an increase in immunosuppressive cytokines and polarization of monocytes into M2 polarized macrophages was found, highlighting the importance of the presence of stromal components in the preclinical models. A simple scaffold-free 3D spheroid model of direct PDAC and PSC co-cultures has been reported to facilitate the study of cellular cross-talk (Norberg et al., 2020). The authors observed a shift in the phenotypes of both the cell populations, tumor cells to be more mesenchymal and the activated pancreatic stellate to a myofibroblast like phenotype, indicating a tumor-stroma crosstalk. They also employed an interesting interspecies approach where human PDAC is co-cultured with mouse PSC to investigating cell-type specific gene expression in intact spheroids, using species-specific primers. In another example, infiltration of NKG2D expressing T cell and NK cell in a primary colorectal spheroid model was seen (Courau et al., 2019). Using this approach, targeting MICA/B molecules of the NKG2D axis resulted in increased NK cell infiltration and cytotoxicity. Collectively, these studies demonstrate the potential advantages of utilizing a simple but reliable 3D model to evaluate therapy response in the setting of immuno-oncology. One limitation of this technique is the dependency on cell self-aggregation, which restricts control over the 3D culture environment, and its defined architecture, what may be critical for systematic investigation of certain TME characteristics as well as their response to drug treatment.

### 4.2 Organoid System

Organoid technology has grown in popularity during the last decade. Organoids are 3D structures formed from tissues with multiple cell lineages, including stem cells and differentiated cells (Delle Cave et al., 2021), which retain the ability to self-renew and self-organize in a mini-organ-like structure that resembles the architecture and the cellular heterogeneity of the tissue of origin (Broutier et al., 2017; Shi et al., 2020). Organoids also preserve the genetic stability of the cells, allowing for improved modelling of tissue processes. Recently, organoid technology has grown in popularity in PDAC research. It is now possible to recapitulate disease-specific alterations *in vitro*, allowing researchers to mimic the various phases of tumor formation (Boj et al., 2015b). In fact, technology has improved to the point that organoids can be grown from very small biopsies, what permits to examine patients with tumors that are localized, advanced, or metastatic as shown for example by Tiriac et al. (2019), in PDAC samples.

Patient-derived organoids (PDO) are generated by embedding a single cell suspension of tumor cells isolated from primary tissue digestion or fine needle biopsy in a matrix such as matrigel or collagen (Gao et al., 2014; Kopper et al., 2019). These matrices are then supplemented with tumor-selective medium containing a well-defined mix of growth factors, such as R-spondin (RSPO), WNT3A, epidermal growth factor (EGF) and bone morphogenetic proteins (BMP) inhibitor Noggin. These factors help stem cells to maintain their ability of differentiation and self-renewal (Sato et al., 2009).

Previously, the use of clonally derived organoids to reveal patient-specific sensitivities to new medicinal drugs has been reported (Huang et al., 2015). A heterogeneous response to EZH2 inhibition in PDOs was observed, which correlated with H3K27me3 expression in both tumor organoids and matched patient tumors, demonstrating organoids’ capacity to maintain the original tumor’s epigenetic signatures. More recently, a well-characterized biobank of 30 patient-derived organoids was used to undertake comprehensive drug screenings, revealing distinct drug sensitivity profiles (Driehuis et al., 2019). These findings highlight the enormous potential of PDOs in precision medicine. Although co-clinical trials in PDO have been described with success in gastrointestinal malignancies and PDAC, in general access to patient tissue is restricted due to the need of performing this type of studies in a safe environment for the patients (clinical trial or similar) and the need of the tissue for a correct diagnosis (Vlachogiannis et al., 2018; Seppälä et al., 2020). Moreover, the success rate of PDO depends on tumor type, amount of starting material (resection versus fine needle aspiration) and treatment history (Busslinger et al., 2020).

As an alternative to PDO, pancreatic organoids generated from wild-type mice and genetically modified mouse models have been demonstrated *in vitro* to precisely replicate physiologically relevant features of PDAC development (Boj et al., 2015a). When compared to PDOs, mouse organoids can be obtained considerably more easily in terms of sample accessibility and amount. Additionally, mouse organoids are far more amenable to various genetic manipulations, facilitating mechanistic studies. Furthermore, results obtained from the organoid model may be easily transferred to an *in vivo* model with the same mutational background in an immunocompetent model. Working with mouse organoids also allows us to study different stages of PDAC development (Boj et al., 2015a). This has resulted in better understanding of the mechanisms underlying the development and progression of PDAC, and it is an excellent tool for the study of immuno-oncology.

Organoid culture represents a novel approach to investigating the immunobiology of PDAC tumors. As previously stated, epithelial-only submerged Matrigel organoids can aid in predicting a patient’s response to therapy and selecting individualized treatment methods; nevertheless, these organoids do not fully recreate the TME due to the absence of stromal components. Organoid technology, is fast adapting to incorporate stromal cells, allowing for the research of diverse immunotherapy strategies as well as studying immune evasion (Bar-Ephraim et al., 2020; Fitzgerald et al., 2020).

#### 4.2.1 Reconstitution of Stromal Components

Organoids may now be co-cultured with exogenously provided stromal components such as cancer-associated fibroblasts (CAFs) and immunological populations, including peripheral blood mononuclear cells (PBMCs), leukocytes, tumor-associated macrophages (TAMs), and DCs (Boucherit et al., 2020). These stromal cells can be isolated either from the tumor fragments (i.e., Tumor infiltrating lymphocytes) or from peripheral blood mononuclear cells (PBMCs). Co-culture of human PDAC organoids with pancreatic stellate cells, a precursor population of CAFs, has led to better understanding of CAF heterogeneity. A pioneer study by Öhlund et al. (2017) using a tumor organoid-CAF co-culture revealed the presence of two spatially separated, mutually exclusive, dynamic, and phenotypically distinct CAF subtypes: alpha-SMA expressing myofibroblasts (myCAFs) and inflammatory cytokine secreting CAFs (iCAFs). Another study reported that squamous *trans*-differentiation in an aggressive p63-expressing squamous PDAC was associated with neutrophil infiltration and other markers of inflammation (Somerville et al., 2020). Using *in vitro* PDAC organoid models and *in vivo* mouse models, the authors discovered p63-induced IL-1 secretion which in turn promotes iCAF formation. It is apparent that such co-culture models are an excellent tool for understanding CAF heterogeneity, which is an important factor to consider when designing stroma targeting immunotherapies.

A well characterized multi-cell type organotypic co-culture model of the tumor microenvironment has been reported (Tsai et al., 2018). Human PDAC organoids grown with matched tumor-associated fibroblasts and immunological components of the tumor microenvironment reveal the emergence of a sophisticated disease-representative model. In a recent study, Freed-Pastor et al. (2021) demonstrate the use of mouse models and organoid/CD8^+^ T cell co-culture system to model neoantigen expression. The use of organoid co-culture offered flexibility and genetic tractability to investigate new and diverse neoantigens. Using two such complementary model systems, the authors identified a central role of CD155/TIGIT axis in mediating immune evasion in PDAC (Freed-Pastor et al., 2021). Using a more complex cellular set up, Chakrabarti et al. (2018) reported the use of a co-culture of gastric tumor organoids from mouse models with cytotoxic T lymphocytes (CTLs) and bone marrow-derived DCs, to potentially predict the efficacy of immune-checkpoint inhibition for the treatment of gastric cancer. They observed an increase in CD8 lymphocyte mediated cell killing with the inhibition of PDL1. This indicates that this approach may also be extended to PDAC as a platform for the study of immunotherapy (Chakrabarti et al., 2018). Along the same lines, a recent study demonstrated the use of murine and human pancreatic ductal adenocarcinoma (PDAC) autologous organoid-immune cell co-culture to test efficacy of a combinatorial immunotherapy involving PD-1 inhibition and MDSC depletion (Holokai et al., 2020). The authors observed that PDAC co-culture with MDSCs promoted tumor growth and suppressed T cell proliferation, and when treated with the combination therapy rendered the organoids susceptible to anti-PD-1/PD-L1-induced cancer cell death. This demonstrates the value of pre-clinical organoid models in predicting the success of targeted therapies to enhance patient outcomes. However, such a model has its disadvantages, most notably the lack of native stromal components. Immune cells are isolated from blood that has not been constantly exposed to tumor antigens. Also, obtaining pure populations of primary cells on a regular basis from matched donor is relatively more difficult, especially when isolating low abundance cells. Another important factor to consider is the cell culture medium used. Organoid media contains very particular growth factors that may influence T cell activity. More research into the effects of each component on T cell activity and optimizing culture conditions would be extremely advantageous to set up appropriate co-culture systems.

#### 4.2.2 Air-Liquid Interface

Air-liquid interface (ALI) 3D culture was originally reported by the Kuo lab in 2009, who described an application of this approach for a sustained 3D *in vitro* intestinal epithelial culture (Ootani et al., 2009). Later, this strategy was adapted to model PDAC-tumor immune microenvironment using patient derived organoids (Neal et al., 2018). To establish an ALI culture, minced primary tissue fragments are embedded in a collagen gel in a compartmentalized chamber with a porous membrane, similar to a transwell dish, to physically separate from the underlying medium (Yuki et al., 2020). The culture media in an outer dish diffuses into the inner dish via the permeable transwell, while the top of the collagen layer is exposed to air *via* an ALI, providing cells access to an adequate oxygen supply. Additionally, this system allows for vigorous expansion of primary epithelium for a long-term culture as organoids with multilineage differentiation, including endogenous native stromal and immune components *via* improved oxygenation *in vitro* (Li et al., 2016). This technique successfully retains the original tumors’ genetic alterations as well as the TME’s complex cellular composition and architecture. This method allows primary pancreatic ductal epithelium to be cultured in close apposition to myCAFS and iCAFS, known to be antitumorigenic and pro-tumorigenic respectively, hence influencing PDAC growth *in vitro* (Neal et al., 2018). Furthermore, the potential inclusion of all immune components, including tumor-infiltrating lymphocytes and myeloid cells, makes this a suitable system for precision medicine to examine patient-specific drug response.

PDAC ALI 3D culture is a relatively new technique, with only one published report on establishment and characterization of the culture system. Hence extensive study is required to understand its full potential in immuno-oncology. As it is, a major drawback of this technique is the inability to monitor changes in real-time of the cellular and molecular features. Moreover, this culture system is less compliant to genetic manipulation due to its “*en bloc*” nature.

Although the organoid models described above reproduce the complexity observed in the 3D tissue architecture of living organs to a certain extent, they fail to incorporate the mechanical forces that can substantially influence cancer cell behavior, for instance, fluid shear stress and hydrostatic pressure. Furthermore, neither of these systems model a functional vasculature which is perfused by nutrient-rich medium, which results in an inability to study recruitment of circulating immune cells and also bioavailability of the test therapeutic agents.

### 4.3 Microfluidic System

Microfluidic culture, also known as organ-on-a-chip, combines the benefits of 3D culture in a dynamic and controlled environment. This system can be utilized to examine many aspects of carcinogenesis, as well as to perform drug screening and forecast response treatments. Both spheroids and organoids have been reported to be used in microfluidic systems. In essence, the 3D culture is deposited in a microfluidic chip, and the medium is dynamically perfused with or without therapeutic drugs, this allows to offset many of the limitations mentioned above. Additionally, microfluidic methods provide a set of unique capabilities for real-time monitoring of cellular processes, which is critical for the investigation of dynamic tumor-stroma interactions (Zervantonakis et al., 2012). Furthermore, the technology may now be scaled to achieve a larger throughput, increasing the prospect of precision medicine (Schuster et al., 2020). In this study, the authors describe an automated, high-throughput, microfluidic PDAC PDO platform to screen combinatorial and dynamic drug treatments on hundreds of cultures. This integrated platform designed to mirror real patient treatment, combined with real-time analysis of organoids has a great potential in the field of immuno-oncology.

A microfluidic co-culture of pancreatic tumor spheroids with stellate cells has been reported to investigate epithelial to mesenchymal transition and drug resistance (Lee et al., 2018). They embedded 3D tumor spheroids and PSCs separately in type 1 collagen and loaded into each designated channel with intermittent feeding of media. With this system, activation of PSCs under co-culture conditions which in turn influenced the migratory ability of cancer cells was observed. There are multiple reports on microfluidic systems of tumor-immune cell co-culture to study cytotoxic activity as well as resistance to immunotherapy in a 2D system (Aref et al., 2018; Rothbauer et al., 2018). In comparison, 3D models in a microfluidic system are a relatively recent advance. In an interesting proof of concept study reported by Aung et al. (2020), the development of a multicellular tumor-on-a-chip platform was described. Herein, the authors studied the effect of the tumor microenvironment, especially the presence of monocytes and hypoxia have on breast cancer spheroids. They observed increase recruitment of T cells in the hypoxic condition. Moreover, the addition of monocytes to the cancer cells improved T-cell recruitment. Highlighting their potential application in studying recruitment of cells by the tumor cells. A study led by Jenkins demonstrated the use of an organotypic 3D microfluidic culture from murine and patient derived tumor tissue, which retained the native immune TME, in assessing response and resistance to immune checkpoint blockade (Jenkins et al., 2018). They discovered that targeting the TBK1/IKK axis improved responsiveness to PD-1 blockade using their model system, highlighting its application in immuno-oncology. An organoid co-culture in a blood-perfusable pericyte-coated microfluidic chamber has also been reported (Palikuqi et al., 2020). In this system, the endothelial cells could functionally arborize patient derived colorectal cancer organoids and respond to microenvironment stimuli. A system like this has a lot of potential as a tissue-specific *in vitro* platform for the evaluation of administration and response to modified immune cells like CAR-T cells and chemotherapeutic drugs.

Microfluidic systems heavily rely on microfabrication technologies. Such resources and the expertise are not readily available to all researchers. Moreover, this technique also suffers from the same limitations as organoid system with respect to the cells/tissues used. The decision to adopt any of the given model systems should be strongly based on the research objective and the system providing benefits that would outweigh the limitations.

## 5 Conclusion and Future Directions

Our understanding of PDAC tumor biology and the tools used for its research have advanced significantly over the years, however, patients’ prognosis remains very poor. A rational treatment strategy that considers the intricate tumor cell-cell and cell-ECM interactions, as well as the tumor-drug interaction may help to improve this adverse scenario. 3D models may offer a great deal of promise as surrogate tumor models, but their choice should be strictly based on the question under investigation.

To improve the predictive response of a model system for studying treatment response, one factor to consider may be tumor subtype. As previously stated, PDAC has multiple subtypes, each with a varied response to different treatments. In fact, the basal subtype is shown to have an altered metabolism favouring glycolysis (Daemen et al., 2015) and a high lactate content in these tumors might impair the immune response and cause treatment resistance (Husain et al., 2013; Manoharan et al., 2021). Hence, these factors should be included in the 3D model systems used for identification of novel drug sensitivities and resistance mechanisms in PDAC. To date, no reports of immunotherapy responses stratified by tumor subtype have been published. 3D cultures might be an alternate method of reanalysing current preclinical data. The use of PDAC patient-derived organoids that represent each subtype should be a suitable foundation for creating valid models for molecular characterisation and response prediction.

In addition, a predictive model should consider the complex tumor-stroma interaction and future therapeutics should be targeted towards the pro-tumorigenic population. For example, of the various fibroblast subtypes present in the tumor mass, targeting the inflammatory subtype found distal to the tumor is beneficial, whereas targeting the SMA positive population adjacent to the tumor mass is counterproductive. Diffusible small molecule metabolites may be involved in the interaction of tumor cells with distant stromal cells. Thus, an innovative approach that combines an efficient 3D model with technologies to resolve the role of soluble factors, such as mass spectrometry imaging-based metabolomics, may be uniquely suited to elucidate this phenomenon. Combining an optical image of a co-culture containing the desired cellular population with an averaged mass spectrum of the sample’s molecular components, the spatial distribution of metabolic signals involved in the bidirectional communication may be visualized and a proper understanding of the cellular crosstalk may be achieved.

Several clinical trials are now underway in PDAC that employ immunotherapy combinations with standard-of-care chemotherapy (Desai et al., 2019; Bockorny et al., 2020). The design of clinical trials should be guided by solid preclinical data that highlight optimal synergistic combinations to boost anti-tumor immune activity in order to get the best possible outcome. Current chemotherapy regimens need a high dosage which in addition to the apparent toxicity may have a negative effect on the overall immune function. A preclinical study using the 3D models to determine lower concentrations of these drugs that could synergize with immunotherapy would be extremely beneficial. Using an array of organoids that differentially express immune checkpoint inhibitors to screen for effective ICI-Chemotherapy combinations may result useful for designing treatment strategies. A well-characterized 3D model system integrating all aspects aforementioned would stand as an ideal proxy to determine the drug combination that is most effective. Furthermore, it could potentially be utilized to create a therapy-resistant model in order to better understand the mechanism of action.

Another way to look at leveraging 3D models is to generate a set of predictors that can assist in clinical decision making. For example, integrating data from genetically and multi-omically defined PDOs treated with a spectrum of immunochemotherapy combinations, with medical imaging obtained at diagnosis, may possibly uncover predictors of therapeutic response. Large multi-center level studies may aid to facilitate this relevant task.

Given the growing body of evidence supporting the importance of the tumor microenvironment in immune evasion and immunotherapy failure, further research is necessary to fully elucidate the crosstalk between tumor and stroma. 3D models are a tremendous advance and could be used to gain improved understanding of the tumor supportive, immune evading role of stroma. Based on the continued integrative profiling of PDAC, it is likely that the repertoire of 3D models that accurately depict PDAC pathology will expand and be integrated into the treatment decision making process in the next years.
